# Breast Milk Retinol Levels after Vitamin A Supplementation at Different Postpartum Amounts and Intervals

**DOI:** 10.3390/nu14173570

**Published:** 2022-08-30

**Authors:** Danielle S. Bezerra, Andressa T. A. de Melo, Kátia C. de A. N. de Oliveira, Karoline Q. M. A. de Araújo, Monalisa S. M. de F. Medeiros, Flávia A. P. S. dos Santos, Jeane F. P. Medeiros, Mayara S. R. Lima, Ana Gabriella C. L. da Silva, Karla Danielly da S. Ribeiro, Roberto Dimenstein, Mônica M. Osório

**Affiliations:** 1 Department of Nutrition, School of Health Sciences of Trairi, Federal University of Rio Grande do Norte, Santa Cruz 59200-000, Brazil; 2 Postgraduate Program in Nutrition, Federal University of Rio Grande do Norte, Natal 59072-970, Brazil; 3 Department of Tocogynecology, Federal University of Rio Grande do Norte, Natal 59012-570, Brazil; 4 Onofre Lopes University Hospital, Federal University of Rio Grande do Norte, Natal 59012-300, Brazil; 5 Januário Cicco Maternity School, Federal University of Rio Grande do Norte, Natal 59012-310, Brazil; 6 Department of Nursing, Federal University of Rio Grande do Norte, Natal 59072-970, Brazil; 7 Department of Health Sciences, Federal University of Rio Grande do Norte, Natal 59072-970, Brazil; 8 Postgraduate Program in Biochemistry, Federal University of Rio Grande do Norte, Natal 59072-970, Brazil; 9 Postgraduate Program in Collective Health, Federal University of Rio Grande do Norte, Natal 59078-900, Brazil; 10 Postgraduate Program in Nutrition, Federal University of Pernambuco, Recife 50670-901, Brazil

**Keywords:** micronutrients, nutritional status, infant and child nutrition, dietary supplements, chromatography high pressure liquid

## Abstract

Maternal vitamin A (VA) supplementation in risk areas for Vitamin A deficiency (VAD) was launched to improve the level of this nutrient in nursing mothers and in their breast milk. This longitudinal and randomized study aimed to evaluate the levels of retinol in breast milk after supplementation with VA in varying amounts (200,000 IU or 400,000 IU) and different postpartum intervals. Women were distributed into four intervention groups and given a single 200,000 IU postnatal dosage of VA at time 0 h (postnatal morning) (G200 0H); a single 200,000 IU dosage of VA in week four (G200 4W); 200,000 IU of VA at time 0 h + 200,000 IU of VA 24 h after the first supplementation (G400 24H); and 200,000 IU of VA at time 0 h + 200,000 IU of VA one week after the first supplementation (G400 1W). Breast milk samples were collected over a 12-week period (0 h, 24 h and 1, 4, 12 weeks post-natal). Retinol levels were determined by high-performance liquid chromatography. The Generalized Estimated Equation (GEE) assessed the different retinol levels. The G200 (0H), G400 (24H), and G400 (1W) groups presented higher retinol levels at 24 h than the G200 (4W) group (*p* < 0.001). The retinol levels of all groups were similar at times 1, 4 and 12 weeks after delivery (*p* > 0.05). Maternal VA supplementation increased retinol levels in the colostrum. Different supplementation dosages or postpartum administration times did not result in added benefit to retinol levels in mature breast milk.

## 1. Introduction

Vitamin A deficiency (VAD) is still considered a nutritional problem during pregnancy, lactation, and early childhood in many regions around the world, especially in developing countries [1]. Infants have greater vulnerability when compared to the general population, since the status of Vitamin A (VA) during the initial phase of life is influenced by their hepatic stores of retinol at birth, which are usually low, the consumption of AV through breast milk and other foods and by the losses due to infections and parasites [2,3]. In addition, inadequate VA status of the mother can negatively affect their VA concentrations in milk and, consequently, the status of the baby. Thus, even in exclusive breastfeeding, children under two years of age are susceptible to VAD when their mothers produce breast milk with low concentrations of VA, because of their own nutritional deficiency [3].

Maternal VA supplementation in risk areas for VAD was launched as a public health strategy to improve its levels in nursing mothers and consequently to increase VA levels in breast milk [4]. In this sense, in 1997 the World Health Organization (WHO) recommended giving mothers 200,000 IU of VA orally immediately after delivery wherever VAD was considered a public health problem [5]. Once theoretical studies suggested that two dosages could provide a prolonged and safe benefit to the mother-child VA status, the recommendation regarding maternal supplementation was updated in 2002 by the International Vitamin A Consultative Group (IVACG) to two dosages of 200,000 IU, totaling 400,000 IU. The first dosage was indicated to be given immediately after delivery, and the second between 24 h and 6–8 weeks after delivery, which is during the safe, sterile postnatal period [2,6,7].

An interval greater than 24 h between dosages would theoretically be more advantageous because it would ensure a higher retention of the second supplement [8]. The supplementation time could be critical for breast milk VA levels since a second VA supplement given at an optimal time would hypothetically be better stored, helping to benefit the VA status of the mother-child dyad, especially by the increasing of the concentration of VA in mature milk, since colostrum naturally has more retinol [9]. However, no study has investigated whether the interval between dosages would affect the results. Studies until now were designed to assess the benefits of different supplementation protocols, and the expected efficacy of the second supplement has not been confirmed [10,11,12].

The WHO no longer recommends giving VA to women after delivery as a public health intervention to prevent maternal and child morbidity and mortality and reinforces that women after delivery should have an appropriate intake of dietary VA from a healthy and balanced diet [13]. In addition, the WHO 2011 guideline made recommendations for future research, including the need to evaluate the impact of vitamin A (200,000 IU) exactly at the sixth week postpartum compared to early supplementation for lactating women on breast milk retinol concentrations. Moreover, the metabolic aspects and body distribution (hepatic reserves and secretion through breast milk) after the administration of the high doses were also cited as priorities for further studies [13].

Women living in risk areas for VAD may not get adequate amounts of dietary vitamin A to prevent this nutritional insufficiency [14]. In this perspective, the Brazilian Ministry of Health developed in 2005 the “National Vitamin A Supplementation Program”, including maternal VA supplementation (200,000 IU) at the immediate postpartum period in Brazilian areas considered at risk for VAD [14]. However, in 2016 this measure of supplementation for puerperal women was discontinued in the country [15]. However, the postnatal supplementation of mothers with inadequate VA intake remains a way to increase maternal and child (through breast milk) VA stores in the short term [16]. 

Our hypothesis is that supplementation with varying doses of VA administered at different times after parturition will cause varying responses in VA concentrations in breast milk. Bearing in mind the already suggested different maternal supplementation protocols and the existing gaps, this study aimed to evaluate the levels of retinol in breast milk after supplementation with VA in varying amounts (200,000 IU or 400,000 IU) and different postpartum intervals (0 h, 24 h, 1 week and 4 weeks). 

## 2. Materials and Methods

### 2.1. Study Type and Population

The present study is a longitudinal and randomized clinical trial of healthy and low-income women attended at a Brazil public maternity hospital. The study was registered on the Registro Brasileiro de Ensaios Clínicos—ReBEC (number: RBR-5xgjwm) and was conducted from July 2012 to July 2014.

### 2.2. Sample Size Calculation

Sample size was calculated by the inferential method using mean values and measuring the dispersion of breast milk retinol levels, considering that the standard deviation of retinol in breast milk would not exceed 0.43 µmol/L (mean = 3.9 µmol/L) [17]. Consequently, 14 women per group would be necessary to detect a difference of 0.3 µmol/L with a power of 80% and a confidence of 95%.

### 2.3. Participant Recruitment

The participants were recruited for the study at admission for delivery in a public maternity hospital that provides care to women from the municipality of Santa Cruz-Rio Grande do Norte-Brazil and nearby cities. Women aged between 18 and 40 years, with low obstetric risk, primiparous or multiparous, with a full-term singleton pregnancy (>37 weeks) were included in this study.

The exclusion criteria were women aged more than 40 years; women with diabetes, high blood pressure, neoplasms, heart disease, syphilis, human immunodeficiency virus (HIV) and other infectious diseases, gastrointestinal tract diseases, mental disorders, multiple or preterm (<37 weeks) fetuses, cerebral palsy, metabolic disorders, palatal cleft, digestive tract malformations, and lung disease; and women who took VA supplements during pregnancy.

### 2.4. Study Design (Groups and Follow-Up)

The participants were placed in one of the four study groups immediately after delivery. The participants then met periodically with the research team during the next twelve weeks. A 12-week follow-up time was chosen because the first three or four months of lactation seem to be the period of greatest breast milk nutrient variation. Nutrient levels then seem quite stable after this period until the involution of the mammary gland [8,18]. 

In the data collection period, the Supplementation Program of the Brazilian Ministry of Health [14] determined a supplementation (VA) should be given postnatally to all women seen in public hospitals in some Brazilian regions, therefore ethics prevented the inclusion of a non-supplemented group in the present study. 

To compare different doses of vitamin A supplementation and verify the effect on retinol levels in breast milk, we created four intervention groups:

- G200 (0H) = Single VA supplementation: 200,000 IU given orally immediately after delivery;
- G200 (4W) = Single VA supplementation: 200,000 IU given orally four weeks after delivery;
- G400 (24H) = Double VA supplementation: 200,000 IU given orally immediately after delivery + 200,000 IU given orally 24 h after the first supplement (total of 400,000 IU of VA).
- G400 (1W) = Double VA supplementation: 200,000 IU given orally immediately after delivery + 200,000 IU given orally one week after the first supplement (total of 400,000 IU of VA).

**Figure 1:**
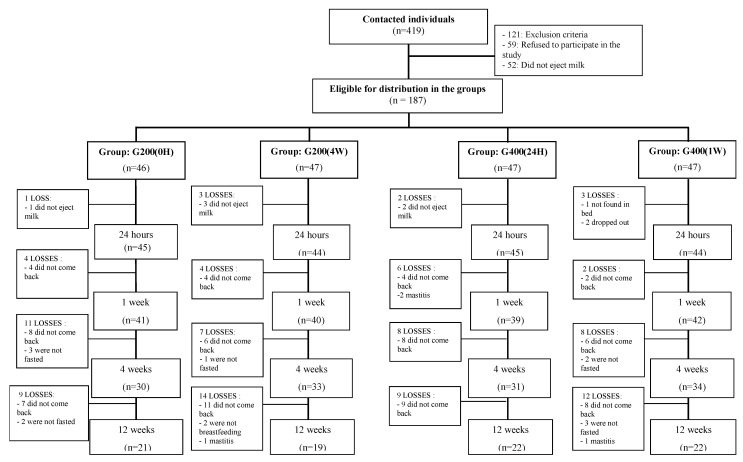
The allocation of patients by supplementation groups and follow-up times. G200 (0H) = 200,000 IU given orally immediately after delivery; G200 (4W) = 200,000 IU given orally four weeks after delivery; G400 (24H) = 200,000 IU given orally immediately after delivery + 200,000 IU given orally 24 h after the first supplement (total of 400,000 IU of VA). G400 (1W) = 200,000 IU given orally immediately after delivery + 200,000 IU given orally one week after the first supplement (total of 400,000 IU of VA).

Figure 1 shows the allocation of participants by supplementation groups and follow-up times.

### 2.5. Intervention and Breast Milk Collection

The first breast milk sample (baseline) was collected before breakfast in the ward the morning after delivery. Breast milk was collected by manually expressing one breast at least two hours after the last nursing. Later, all mothers except those in the G200 (4W) group were given orally one 200,000 IU tablet of VA. 

Then, a new sample was collected from all participants 24 h after the first breast milk collection, followed by the second oral administration of 200,000 IU of VA to the participants in the G400 (24H) group, totaling 400,000 IU of retinol. 

The puerperal women returned to the hospital for infant examination one week after delivery, according to the postnatal follow-up schedule. A new breast milk sample was collected at this time from all participants who showed up, followed by giving the second oral 200,000 IU VA supplement to the participants in the G400 (1W) group, totaling 400,000 IU of retinol.

New breast milk samples were collected during scheduled hospital visits and confirmed by telephone four weeks after delivery. After milk collection in the fourth week, the women in G200 (4W) were given their first and only 200,000 IU VA tablet. The last milk collection of all participants took place on the 12th week after delivery, in previously scheduled home visits.

The VA tablets used by the study contained 200,000 IU (60 mg) of retinyl palmitate + 40 mg of vitamin E [14]. No typical adverse events (vomiting, fontanelle bulging, etc.) of maternal or child poisoning with megadoses of VA were identified or reported. The mothers were strongly encouraged to maintain exclusive breastfeeding.

### 2.6. Anthropometric Parameters

Maternal weight and height collected at the last prenatal visit were taken from the mothers’ follow-up card. The mothers’ nutritional status was analyzed according to the body mass index (BMI), BMI = [mass (kg)]**/**[height (m)]^2^ for gestational age proposed by Atalah, Castillo, Castro and Aldea [19] and adopted by the Brazilian Ministry of Health [20]. 

The infants’ weight and length were collected from their hospital records and analyzed according to their z-score by the Anthro version 3.01 (WHO, Anthro 2009, Geneva, Switzerland) software program [21].

### 2.7. Other Variables of Interest

The following delivery-related information was collected from the patients’ medical records to characterize the sample: parity, delivery route, and neonatal sex. The participants were also interviewed to collect the following sociodemographic data: maternal age, marital status, residence location, number of individuals in the household, maternal education level, and family income.

### 2.8. Biochemical Retinol Assessment

The samples were placed in sterile polypropylene tubes wrapped in aluminum foil to protect the samples from light. The samples were then frozen to −20 °C and taken to the Laboratory where they were quantified and analyzed.

Milk samples were extracted for retinol analysis by adapting the method developed by Giulliano, Neilson, Kelly and Canfield as described below [22]. 

After thawing the samples in water baths ≤37 °C under 60 rpm stirring, the samples were submitted to alkaline saponification with two sample volumes of 50% *v*/*v* potassium hydroxide (Vetec^®^, Rio de Janeiro, Brazil) to hydrolize the retinyl esters. One sample volume of 95% ethanol (Vetec^®^, Rio de Janeiro, Brazil) was also added to the sample to denature the proteins. Next the samples were homogenized for one minute by a vortex mixer and submitted to a 45 °C warm bath for two hours.

Two milliliters of the extraction reagent hexane (Merck^®^, Darmstadt, Germany) were added to the samples, followed by one minute of vortex mixing and centrifugation at 4000 rpm for 10 min. The supernatant (hexane phase) was placed in another tube, where another 2 mL of hexane was added. This procedure was repeated three times. 

A 37 °C water bath was used for evaporating approximately 3 mL of the hexane phase in a nitrogen atmosphere, and the resulting extracts were diluted again in 1 mL of high-performance liquid chromatography (HPLC)-grade methanol (Vetec^®^, Rio de Janeiro, Brazil). After stirring the samples in a vortex mixer for one minute, 20 μL aliquots were placed in a chromatographer (Shimadzu^®^, Kyoto, Japan) to quantify retinol. 

The mobile phase used for the analysis was methanol at 100% in an isocratic system with a flow of 1 mL/min and 4.2-min retention time. Absorbance was monitored at a wavelength of 325 nm. Retinol in the samples was identified and quantified by comparing the area under the chromatographic peak with retinol’s standard area under the peak (Sigma-Aldrich^®^, St. Louis, MO, USA). The concentrations of the standards were confirmed by an extinction coefficient specific for retinol in absolute ethanol (ε 1%, 1 cm = 1.780 at 325 nm).

Retinol in breast milk is presented in µmoL/L and used for expressing the nutritional status of maternal VA [23]. Values < 1.05 µmoL/L were considered as indicative of subclinical VAD in breast milk [24].

### 2.9. Statistical Analyses

Descriptive data analysis was performed using the statistical package IBM SPSS Statistics for Windows version 22.0. Armonk, NY: IBM Corp . Continuous variables were expressed as means (±standard deviations or 95% confidence interval) and the categorical variables as percentages (%). In order to investigate the similarities between the characteristics of the experimental groups, the inferential part used Pearson’s chi-squared test (χ^2^) and one-way analysis of variance (one-way ANOVA) for comparing proportions and means, respectively. A generalized estimating equation (GEE) with linear link function, autoregressive working correlation matrix (AR-1), Wald test for testing hypothesis, and Bonferroni correction investigated the effects of the independent variables (maternal group, follow-up time, living location, family income, education level, and anthropometric nutritional status) on breast milk retinol levels. The significance level was set at 5% to minimize type-I error.

## 3. Results

The study participants had a mean age of 25.5 ± 4.9 years, lived in the urban areas (79%) of a municipality in Brazil, were married or living with their partners (86.4%), had incomplete elementary or high school (65.3%), and had a monthly family income equal to or below one Brazilian minimum monthly salary (61.7%) (Table 1). At the end of pregnancy, 42.4% of the participants had normal gestational BMI (Table 1). Their mean parity was 1.04 ± 1.2 and most performed vaginal delivery (75%) (Table 1).

The mean gestational age of the neonates was 37.3 weeks; most were males (54.8%), and most had normal nutritional status (96.4%) according to the weight-for-age (W/A) ratio (data not shown). Moreover, 95.2% of the infants were still being exclusively breastfed 12 weeks after delivery, and only 4 infants had been introduced to water and/or tea (G200 0H: *n* = 2; G400 24H: *n* = 1; G400 1W: *n* = 1) (data not shown).

The characteristics of the women and infants in the four experimental groups were similar (*p* > 0.05) (Table 1).

The predictive model showed that the follow-up time and the interaction group-follow-up time had a significant impact on breast milk retinol levels (*p* < 0.001) (Table 2).

Table 3 shows the mean retinol levels in the breast milk of the four treatment groups at the various follow-up times. The Bonferroni post hoc correction identified changes in the breast milk levels of retinol in the different treatment groups (*p* < 0.05) (Table 3).

**Figure 2:**
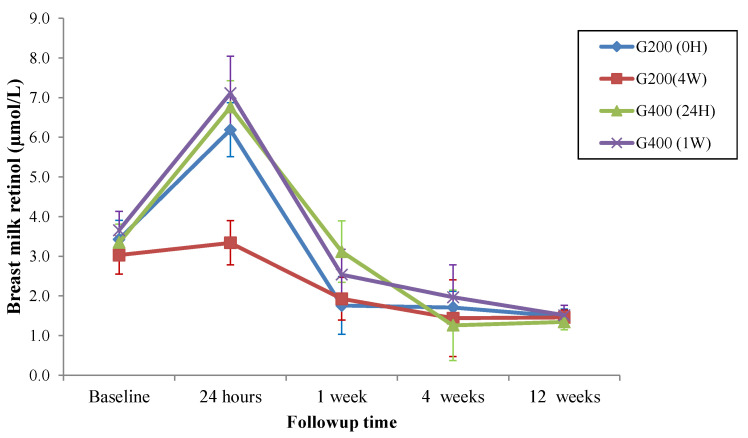
An intergroup assessment of breastmilk retinol levels by follow-up time, Brazil. G200 (0H) = 200,000 IU given orally immediately after delivery; G200 (4W) = 200,000 IU given orally four weeks after delivery; G400 (24H) = 200,000 IU given orally immediately after delivery + 200,000 IU given orally 24 h after the first supplement (total of 400,000 IU of VA). G400 (1W) = 200,000 IU given orally immediately after delivery + 200,000 IU given orally one week after the first supplement (total of 400,000 IU of VA). Bonferroni post hoc correction with statistical significance for *p* < 0.05. G200 (0H) *n* = 21; G200 (4W) *n* = 19; G400 (24H) *n* = 22; G400 (1W) *n* = 22.

All groups had similar baseline levels of breast milk retinol (*p* = 0.83). However, retinol level in the colostrum at 24 h was lower in the G200 (4W) group than in the G200 (0H) (*p* = 0.007); G400 (24H) (*p* = 0.001); and G400 (1W) (*p* = 0.006) groups, which remained similar (*p* = 1.00) at 24 h. All groups had similar breast milk retinol levels at 1 week, 4 weeks, and 12 weeks (*p* > 0.05) (Figure 2).

The nutritional classification of maternal AV status, performed according to the cutoff points adopted for breast milk, identified that 8.3%; 1.5%; 28.1%; 29.4% and 44.0% of all nursing mothers had subclinical VAD at baseline, 24 h, 1 week, 4 weeks, and 12 weeks postpartum, respectively.

## 4. Discussion

This study aimed to assess retinol levels in breast milk after vitamin A supplementation in different amounts and postpartum intervals. The present study used breast milk since breast milk can show the effectiveness of VA-related interventions and help to monitor VA status [16,24]. Moreover, breast milk is not affected by systemic inflammation [25].

Assessment of the effect of different VA supplementation protocols on the breast milk retinol levels showed that maternal VA supplementation increased retinol levels in the colostrum, but different supplementation dosages or postpartum administration times did not result in an added benefit to retinol levels in mature breast milk.

All four groups had similar levels at baseline, 1 week, 4 weeks, and 12 weeks after delivery. Hence, although other studies have not assessed the ideal interval for maternal supplementation, the present results are similar to other published results: maternal supplementation in the postpartum period increases retinol concentrations in breast milk [26,27,28] in the short term, being especially relevant when the mother’s previous concentrations are low [28]. Added to this is the fact that retinol levels in different body fluids do not differ after maternal supplementation with varying megadoses of VA [10,11,12,29]. 

The apparent absence of a demonstrable effect from the different supplementation protocols on breast milk retinol levels does not necessarily mean that postnatal maternal VA supplementation is not beneficial and necessary. The most important question is whether the use of megadoses of VA would be enough to meet the infant’s basic requirements [30] and to create hepatic stores for the critical breastfeeding period. 

The mean breast milk retinol levels at different assessment times showed that all groups were above the cut-off point for VAD in breast milk (1.05 µmol/L) [24]. However, only the G400 (24H) and G400 (1W) groups had adequate breast milk retinol levels to create VA stores in the infant’s liver (≥2.3 µmol/L) one week after delivery [16]. Moreover, all groups had inadequate breast milk VA to create hepatic stores at weeks 4 and 12. This is concerning because children under two years of age mostly depend on breast milk to get VA and create the hepatic VA stores they need after weaning [16]. Thus, the study infants are at risk of VAD because they already cannot obtain enough retinol to create hepatic stores in the exclusive breastfeeding period. 

Furthermore, since the analysis of mean retinol concentrations of the maternal population may mask the proportion of women with retinol below or above the cutoff point, and as the prevalence of subclinical VAD increased in the population during the lactation segment, this could indicate that the administered dose would not be sufficient for some mothers during the lactation period. However, as pointed out by the same research group of the current study, maternal supplementation with high doses of VA deserves caution because it may negatively interfere with the bioavailability of other nutrients such as α-tocopherol, which may harm the health of the newborn, as newborns have limited reserves of vitamin E [31]. Therefore, these patients require monitoring during pregnancy, as well as throughout lactation, as they may be exposed to risk factors for themselves and their babies. 

The groups that received at least one megadose of VA immediately after delivery (G2000H, G40024H, G4001W) had significantly higher colostrum VA levels at 24 h than at baseline (*p* < 0.05). This increase was not seen in the G200 (4W) group (*p* > 0.05) since the participants in this group only received the supplement four weeks after delivery. Hence, it can be suggested that supplementation immediately after delivery is quantitatively more advantageous than supplementation at four weeks because the naturally high colostrum retinol levels [29] increased in the G200 (0H), G400 (24H), and G400 (1W) groups, thereby maximizing and anticipating the benefit to the maternal and child nutritional status. Additionally, maternal VA supplementation immediately after delivery may also increase the colostrum immunoglobulin A (IgA) levels [8]. This factor is promising with respect to the potential benefits to the infant’s health and survival and reinforces the importance of feeding the newborn with maternal colostrum [30]. Like VA levels, IgA levels tend to decrease as the milk matures [8,29]. Therefore, these facts make the period immediately after birth a key time for maternal supplementation/nutrition considering the improvements in the nutritional status and immunity of mothers and infants. 

Since the lactation stage (colostrum, transition milk, and mature milk) impacts VA level in breast milk [29], it would be important to find a strategy capable of keeping retinol levels high as much as 12 weeks after delivery. However, a comparison of the treatment protocols showed that even mothers given the supplement 4 weeks after delivery (G200 4W) did not have higher breast milk retinol levels than the other groups in week 12. Yet, retinol levels in weeks 1, 4, and 12 were higher than the cut-off point for VAD (1.05 µmol/L) and similar in all groups (*p* > 0.05), which is certainly favorable for the nutritional status of mothers and infants. Moreover, the eight-week interval between the last two measurements (weeks 4 and 12) may have been too big to detect differences in the retinol levels of the G200 (4W) group and the other groups. 

The group which received two supplements with a 24-h interval (G400 24H) had higher retinol levels than the other groups at week one, but the difference was not significant (*p* > 0.05). Retinol levels in week one could have increased due to the second supplementation 24 h after delivery, but this was not enough to cause significant differences between the groups. Thus, the G400 (24H) protocol could have affected the natural decreasing trend of retinol in breast milk, making the retinol levels of transitional milk closer to the baseline levels. However, this similarity was not seen in weeks 4 and 12 after delivery (*p* < 0.05), when VA concentrations in mature breast milk declined.

The use of a double supplementation protocol, even in the immediate postpartum period (G400 24H), for the short-term prevention of VAD in populations at risk would be logistically more viable than one supplement given four weeks after delivery (G200 4W) or the double supplementation with a one-week interval (G400 1W). The smaller interval (G400 24H) would allow the women to receive the two supplements at the hospital, so a second visit would not be required for a first (G200 4W) or second (G400 1W) dosage. However, there was no additional effect of 400,000 IU VA on breast milk retinol levels at the end of the segment (12th week) when compared to a standard dose of 200,000 IU after delivery, similar to what was found by Rajwar et al. (2020) in their systematic review and by Tomiya et al. (2015) in their randomized clinical trial [29]. Furthermore, protocols with supplementation scheduled for after hospital discharge would require the mothers to return to the hospital or home visits by the healthcare team, which sometimes is not possible because of great distances and/or difficult access. To address these issues, daily oral supplementation with low doses of VA may be useful to improve maternal VA status, although the effect on infant health status through breast milk has not been proven [32]. Routine VA supplementation from the integration of child and reproductive health services may also be considered an appropriate strategy, improving complementary feeding practices and access to family planning [33]. Furthermore, the G400 (24H) protocol was statistically similar to the G200 (0H) protocol in all occasions, and numerically inferior in weeks 4 and 12 after delivery. These protocols (G400 24H and G400 1W) also presented the additional disadvantage of increasing costs, making selection difficult. 

Nevertheless, these data should be interpreted with caution since the present study was conducted in a single maternity hospital located in a poor municipality of Brazil, limiting extrapolation of the results to groups with better socioeconomic status and different characteristics which could influence the results. In addition, another limitation of this study was the small sample size in each group. In any case, since maternal supplementation is a prophylactic and emergency measure, and current evidence presented herein does not support the use of VA megadoses for postpartum women to maintain cumulative increase in VA levels during the infant’s first months of life, it is understood that it is necessary to ensure that scarce resources in economically disadvantaged populations like those in northeastern Brazil are carefully managed and directed to a set of strategies which can increase mother and baby VA status. This should include ensuring that postpartum women have adequate and sufficient intake of VA from a healthy diet, as well as enabling other means to combat VAD such as food fortification and low doses of VA daily or weekly. This may be honorable in the short or medium term, but it will certainly increase the sustainability of the positive results and reduce the long-term costs of all users. This will surely most consistently and decisively improve VA status, overall health, and the quality of maternal and child life. Other similar research should be encouraged and deepened, in order to contribute to a better understanding of the complexity of AV metabolism in lactating women, helping to make decisions.

## 5. Conclusions

Our results point to the need for the adoption of measures to combat and control VAD in the population studied. Colostrum retinol levels increased after immediate postpartum VA supplementation, but different supplementation dosages or administration times postpartum did not result in additional benefit to retinol levels in mature breast milk. Considering the specificities and fragilities of each biological, social, and economic context in which each maternal and infant group is inserted, maternal VA supplementation with a dose of 200,000 IU or less is justified as a prophylactic option for lactating mothers at risk of insufficient VA intake, thus reducing the likelihood of VAD for her and the infant. It is also essential that women are assisted in an educational manner regarding dietary sources of vitamin A throughout pregnancy and lactation, enhancing the benefits related to health education.

## Figures and Tables

**Table 1:** Characteristics of the study participants and infants, Brazil.

Variables	G200 (0H) (*n* = 21)	G200 (4W) (*n* = 19)	G400 (24H) (*n* = 22)	G400 (1W) (*n* = 22)	*p*-Value	Total (*n* = 84)
MATERNAL						
Age (years)	24.6 ± 3.9 ^a^	26.8 ± 5.6 ^a^	24.0 ± 4.5 ^a^	26.7 ± 5.1 ^a^	0.15 ^c^	25.5 ± 4.9
Parity	1.1 ± 1.7 ^a^	0.9 ± 1.1 ^a^	0.9 ± 0.8 ^a^	1.3 ± 1.1 ^a^	0.66 ^c^	1.0 ± 1.2
Marital status					0.84 ^d^	
Single	4 (4.9) ^b^	2 (25) ^b^	3 (3.7) ^b^	2 (2.5) ^b^		11 (13.6)
Married	17 (21.0) ^b^	16 (19.8) ^b^	19 (23.5) ^b^	18 (22.2) ^b^		70 (86.4)
People per household					0.47 ^d^	
Up to three	14 (17.6) ^b^	11 (13.8) ^b^	13 (16.3) ^b^	13 (16.4) ^b^		51 (64.1)
Four or more	7 (8.8) ^b^	7 (8.8) ^b^	8 (10) ^b^	7 (8.8) ^b^		29 (36.3)
Education level					0.48 ^d^	
High school and higher education	6 (7.4) ^b^	5 (6.1) ^b^	8 (9.9) ^b^	9 (11.0) ^b^		28 (34.6)
Up to elementary school	15 (18.5) ^b^	13 (16.1) ^b^	14 (17.3) ^b^	11 (13.6) ^b^		53 (65.3)
Family income (minimum salaries)					0.98 ^d^	
≤1	13 (16.0) ^b^	11 (13.6) ^b^	12 (14.8) ^b^	14 (17.3) ^b^		50 (61.7)
>1	8 (9.9) ^b^	7 (8.7) ^b^	10 (12.3) ^b^	6 (7.4) ^b^		31 (38.3)
Residence location					0.57 ^d^	
Rural	5 (6.2) ^b^	3 (3.7) ^b^	3 (3.7) ^b^	6 (7.4) ^b^		17 (21.0)
Urban	16 (19.8) ^b^	15 (18.5) ^b^	19 (23.5) ^b^	14 (17.3) ^b^		64 (79.0)
Gestational nutritional status					0.57 ^d^	
Low weight	5 (6.3) ^b^	2 (2.5) ^b^	5 (6.3) ^b^	1 (1.3) ^b^		13 (16.3)
Normal weight	9 (11.3) ^b^	7 (8.8) ^b^	6 (7.5) ^b^	12 (15.0) ^b^		34 (42.4)
Excessive weight	7 (8.8) ^b^	8 (10.0) ^b^	10 (12.5) ^b^	8 (10.1) ^b^		33 (41.3)
Delivery route					0.44 ^d^	
Vaginal	16 (19.0) ^b^	12 (14.3) ^b^	16 (19.0) ^b^	19 (22.6) ^b^		63 (75.0)
Caesarian	5 (6.0) ^b^	7 (8.3) ^b^	6 (7.2) ^b^	3 (3.6) ^b^		21(25.0)
INFANTS						
Gestational age (weeks)	39.8 ± 1.1 ^a^	39.8 ± 1.2 ^a^	40.1 ± 1.2 ^a^	38.8 ± 1.4 ^a^	0.89 ^c^	37.3 ± 2.3
Sex					0.05 ^d^	
Female	8 (9.5) ^b^	14 (16.7) ^b^	8 (9.5) ^b^	8 (9.5) ^b^		38 (45.2)
Male	13 (15.5) ^b^	5 (6.0) ^b^	14 (16.7) ^b^	14 (16.7) ^b^		46 (54.8)
Weight (kg)	3357.9 ± 368.3 ^a^	3430.3 ± 454.6 ^a^	3283.6 ± 435.4 ^a^	3410.7 ± 301.9 ^a^	0.62 ^c^	3368.6 ± 389.6
Length (cm)	49.0 ± 1.5 ^a^	49.3 ± 1.8 ^a^	48.2 ± 1.7 ^a^	49.0 ± 1.6 ^a^	0.24 ^c^	48.9 ± 1.7

G200 (0H) = 200,000 IU given orally immediately after delivery; G200 (4W) = 200,000 IU given orally four weeks after delivery; G400 (24H) = 200,000 IU given orally immediately after delivery + 200,000 IU given orally 24 h after the first supplement (total of 400,000 IU of VA). G400 (1W) = 200,000 IU given orally immediately after delivery + 200,000 IU given orally one week after the first supplement (total of 400,000 IU of VA). Brazilian minimum salary = 243.14 United States dollars; ^a^ = means ± standard deviation; ^b^ = *n* (%); ^c^ = one-way ANOVA; ^d^ = χ^2^. Statistical significance for *p* < 0.05.

**Table 2:** A predictive model for the retinol concentration after intervention and control of maternal variables.

Source	Model		
	Wald χ^2^	df	*p*
Group	3.20	3	0.36
Follow-up time	92.71	4	<0.001 *
Group * follow-up time	65.53	12	<0.001 *
Living location	0.50	1	0.47
Family income	1.35	1	0.24
Education level	1.30	1	0.25
Anthropometric nutritional status	1.70	3	0.63

df: degrees of freedom Dependent variable: retinol Model: group, follow-up time, group * follow-up time, living location, family income, education level, anthropometric nutritional status.

**Table 3:** An intragroup assessment of the means retinol levels in breast milk (µmol/L) in different follow-up times, Santa Cruz, Rio Grande do Norte, Brazil.

Follow-Up	Groups			
	G200 (0H) (*n* = 21)	G200 (4W) (*n* = 19)	G400 (24H) (*n* = 22)	G400 (1W) (*n* = 22)
Baseline (IC95%)	3.43 (2.50–4.36) ^a^	3.03 (2.09–3.97) ^d^	3.35 (2.44–4.25) ^f^	3.66 (2.72–4.60) ^i^
24 h (IC95%)	6.19 (4.89–7.50) ^b^	3.34 (2.20–4.47) ^d^	6.76 (5.38–8.14) ^g^	7.11 (5.18–9.05) ^j^
1 week (IC95%)	1.76 (1.04–2.29) ^c^	1.93 (1.24–2.61) ^d,e^	3.12 (1.87–4.37) ^f^	2.54 (1.87–3.20) ^i,l^
4 weeks (IC95%)	1.71 (1.15–2.28) ^c^	1.44 (1.01–1.88) ^e^	1.26 (0.89–1.63) ^h^	1.97 (1.53–2.41) ^l^
12 weeks (IC95%)	1.48 (1.11–1.85) ^c^	1.46 (1.09–1.82) ^e^	1.35 (0.96–1.74) ^h^	1.51 (1.02–2.01) ^l^

G200 (0H) = 200,000 IU given orally immediately after delivery; G200 (4W) = 200,000 IU given orally four weeks after delivery; G400 (24H) = 200,000 IU given orally immediately after delivery + 200,000 IU given orally 24 h after the first supplement (total of 400,000 IU of VA). G400 (1W) = 200,000 IU given orally immediately after delivery + 200,000 IU given orally one week after the first supplement (total of 400,000 IU of VA). 95%CI = 95% confidence interval. Different lower-case letters in the columns = mean intragroup retinol levels differ between the different occasions. Bonferroni post hoc correction with statistical significance for *p* < 0.05.

## Data Availability

Raw data are available upon reasonable request.
